# Vitamin D on Early Stages of Diabetic Kidney Disease: A Cross-sectional Study in Patients with Type 1 Diabetes Mellitus

**DOI:** 10.3389/fendo.2016.00149

**Published:** 2016-12-12

**Authors:** João Soares Felício, Rafael Mendonça Luz, Franciane Trindade Cunha de Melo, Fabricio de Souza Resende, Alana Ferreira de Oliveira, Amanda Soares Peixoto, João Felício Abrahão Neto, Carolina Tavares Carvalho, Denisson Dias da Silva, Marcia Costa dos Santos, Natércia Neves Marques de Queiroz, Manuela Nascimento de Lemos, Elizabeth Sumi Yamada, Karem Miléo Felício

**Affiliations:** ^1^Endocrinology Division, University Hospital João de Barros Barreto, Federal University of Pará, Belém, Pará, Brazil

**Keywords:** type 1 diabetes mellitus, albuminuria, vitamin D, 25(OH)D, diabetic kidney disease

## Abstract

**Context:**

Genetic and environmental factors are involved in the pathogenesis of type 1 diabetes mellitus (T1DM), and vitamin D (VD) deficiency appears as a candidate to risk factor for developing diabetic kidney disease (DKD).

**Objective:**

The purpose of study was to evaluate the existence of an association between low levels of VD and the presence and degree of DKD in T1DM.

**Patients and methods:**

We performed a cross-sectional study, between November 2014 and December 2015. Levels of 25(OH)D and albuminuria were analyzed in 37 patients with T1DM and normal glomerular filtration rate. Thirty-six subjects were evaluated as a control group.

**Results:**

Patients with T1DM and hypovitaminosis D had higher levels of albuminuria compared to those with normal VD levels [albuminuria (log_10_) = 1.92 vs. 1.44; *p* < 0.05]. When we have separated the group of patients according to stage of DKD in patients with normo, micro, and macroalbuminuria, there are lower levels of 25(OH)D in the last when compared to the first two groups (26.7 ± 6.2, 24.8 ± 7.0, and 15.9 ± 7.6 ng/ml; *p* < 0.05, respectively). In T1DM group, we have found correlations between VD levels and both albuminuria and DKD stages (*r* = −0.5; *p* < 0.01 and *r* = −0.4; *p* < 0.05, respectively). A simple linear regression model, with albuminuria as the dependent variable and VD as an independent variable, showed *r*^2^ = 0.2 and *p* < 0.01.

**Conclusion:**

Our data suggest an association between reduced levels of VD and the presence and severity of DKD.

## Introduction

Both environmental and genetics have been known as risk factors involved in the pathogenesis of type 1 diabetes mellitus (T1DM). The vitamin D (VD) deficiency appears as a candidate to risk factor for developing T1DM and diabetic kidney disease (DKD).

The VD deficiency is associated with increased urinary albumin excretion, as well as an increase in the prevalence of cardiovascular disease and mortality in patients with chronic kidney disease in the general population, which has decreased levels of this vitamin (1, 2). The great difficulty in assessing relationship between low levels of VD and DKD is the fact of renal injury itself cause reduced levels of VD. It makes difficult to establish whether VD deficiency would be a triggering environmental factor of DKD or just a consequence of this progression.

A few studies evaluating VD levels in the early stages of DKD and its association with the presence of microalbuminuria have found conflicting results (3–5). Therefore, it is important to establish the existence of a direct relationship between low levels of VD and the presence of DKD in T1DM, particularly in early cases, with normal glomerular filtration rate (GFR).

Therefore, the purpose of the present study was to evaluate the existence of an association between low VD levels with the presence and degree of DKD in T1DM with normal GFR.

## Materials and Methods

### Study Design and Patients

We performed a cross-sectional study, between November 2014 and December 2015, in which levels of 25(OH)D and albuminuria were analyzed. Patients were recruited from Endocrinology Division of the Federal University of Pará, included 37 T1DM with normal GFR (≥90 ml/min/1.73 m^2^) (6) and 36 controls with normal serum creatinine and without comorbidities. The control group was recruited in communities near to University Hospital. Both groups were matched by age and GFR. Exclusion criteria were pregnancy, lactation, individuals with a history of liver disease, use of VD/calcium in the last 6 months, prior concomitant history of metabolic bone diseases, hyperthyroidism or hypothyroidism, and patients. The study was approved by the University Hospital João de Barros Barreto ethics committee – protocol No. 2158/11, and it was in accordance with the standards of the National Health Council. Informed consent was obtained from all patients for being included in the study.

### Clinical and Laboratorial Data

All patients had the following clinical parameters measured: weight, height, body mass index (BMI), and systolic and diastolic blood pressure. Diabetic patients (group 1) and controls (group 2) were submitted to the following laboratory tests: glycated hemoglobin (HbA1c), serum creatinine, 25(OH)D, TSH, free T4, total cholesterol and fractions, triglycerides, and fasting glucose. Albuminuria in three 24 h urine samples was measured only in T1DM. HbA1c was measured by HPLC. The levels of 25(OH)D were measured by chemiluminescence immunoassay (7) and are classified according to their levels: deficiency (<20 ng/ml), insufficiency (20.0–29.9 ng/ml), and normal (≥30.0 ng/ml). The GFR was calculated by formula CKD-EPI (8). The DKD was graded in stages by albuminuria and GFR. Albuminuria was performed by immunoturbidimetry (9), and T1DM was classified according to results in normoalbuminuria (<30 mg/g creatinine), microalbuminuria (≥30 mg/g creatinine and <300 mg/g creatinine), and macroalbuminuria (≥300 mg/g creatinine).

### Statistical Analysis

Categorical variables were described as frequency (percentage), numeric variables with normal distribution were described as mean (SD), and the other as median (minimum–maximum). Chi-square and Fisher tests were used to compare categorical variables. The Student’s *t*-test and Mann–Whitney test were used to compare two groups of numerical variables with and without normal distribution, respectively. To establish correlations between variables, Pearson and Spearman tests were used. The ANOVA test compared more than two groups of numerical variables with normal distribution, and the Kruskal–Wallis test was used to compare more than two groups of numerical variables without normal distribution.

In regard to DKD, the stage, normoalbuminuria, microalbuminuria, and macroalbuminuria, was given as DKD indexes the numerals 0, 1, and 2, respectively. Similarly, in regard to VD levels, the status, normal, insufficiency, and deficiency, was given as VD indexes the numerals 0, 1, and 2, respectively. For clarification, the index was used for statistical analysis.

Additionally, albuminuria values were converted to log base 10 (log_10_) to better analyze the data. A simple linear regression model was analyzed using albuminuria as dependent variable and VD as independent variable. We have also created a regression model (backward stepwise) with albuminuria as dependent variable and age, sex, BMI, SBP, DBP, 25(OH)D, HbA1c, and duration of T1DM to determine the important of those variables as independent predictors. After that, another multiple linear regression model was used to verify if a combination of the variables selected from the backward stepwise model could increase the predictive value to albuminuria when compared to the simple linear regression model using only VD levels. Interferences were represented by hypothesis test with a significance level of 0.05 bilaterally. All information was stored and processed using the software Statistical Package for Social Sciences (SPSS) 21.0 (IBM).

## Results

The clinical and laboratorial characteristics of T1DM and controls are shown in Tables 1 and 2, respectively.

**Table 1:** **Clinical characteristics of T1DM and controls**.

	Sex (M/F)	Age (years)	DT1DM (years)	PAS (mmHg)	PAD (mmHg)	IMC (kg/m^2^)
T1DM (*N* = 37)	13/24	28.6 ± 8.1	13.2 ± 6.6	120.3 ± 13.6	76.1 ± 9.6	24.2 ± 3.9
Controls (*N* = 36)	19/17	25.2 ± 3.0	–	119.1 ± 12.3	74.0 ± 7.9	26.7 ± 5.8
*p*	NS	NS	–	NS	NS	<0.05

*DT1DM, duration of diabetes mellitus; SBP, systolic blood pressure; DBP, diastolic blood pressure; BMI, body mass index; NS, not significant*.

**Table 2:** **Laboratory characteristics of T1DM and controls**.

	HbA1c (%)	Vitamin D (ng/ml)	GFR (ml/min/1.73 m^2^)	Microalbuminuria (mg/24 h)	Microalbuminuria (log_10_ mg/24 h)
T1DM (*N* = 37)	10.3 ± 3.2	24.2 ± 7.4	95 ± 20	236.2 ± 690.7	1.78 ± 0.36
Controls (*N* = 36)	5.2 ± 0.3	25.8 ± 11.2	92 ± 11	–	–
*p*	<0.05	NS	NS	–	–

*log_10_, transformation of microalbuminuria value to log_10_; GFR, glomerular filtration rate; HbA1c, glycated hemoglobin; NS, not significant*.

**Figure 1:**
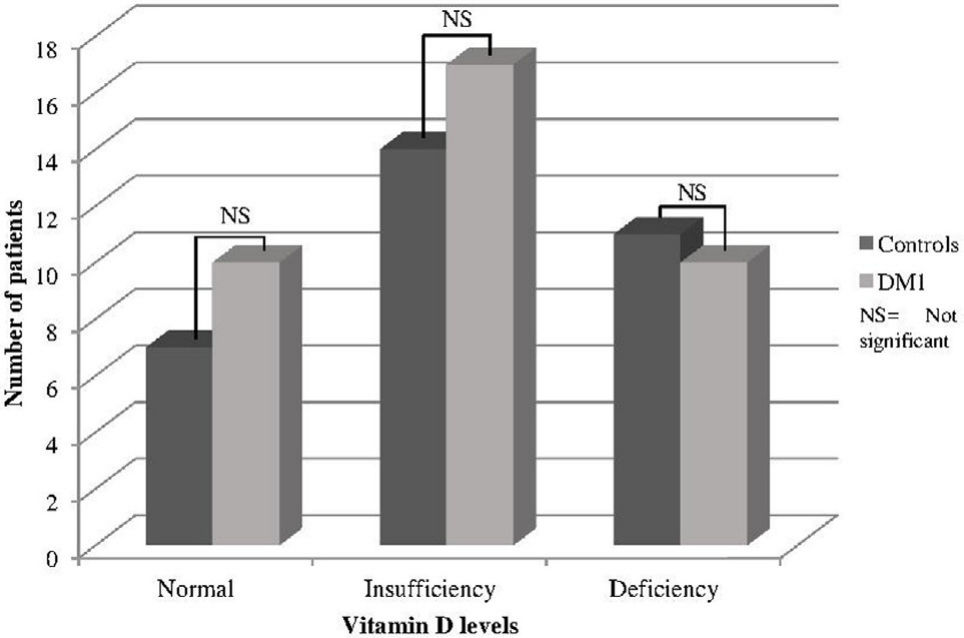
**Status according to VD levels in T1DM and controls**.

There were no differences between T1DM and controls in the number of patients at different stages according to VD levels (Figure 1). The prevalence of hypovitaminosis D among controls was quite high (78%), and there was no difference in relation to T1DM, whose prevalence was 73%. For DKD stage, it was found that 8 patients (21.6%) had normoalbuminuria, 25 patients (67.6%) had microalbuminuria, and 4 patients (10.8%) had macroalbuminuria. The VD levels in different stages of DKD are shown in Table 3.

**Table 3:** **Vitamin D levels and DKD stages in patients with T1DM**.

	DKD stages		
	Normoalbuminuria (mg/24 h)	Microalbuminuria (mg/24 h)	Macroalbuminuria (mg/24 h)
Vitamin D (ng/ml)	26.7 ± 6.2	24.8 ± 7.0	15.9 ± 7.6*

*p < 0.05 vs. normoalbuminuria and microalbuminuria.

Patients with T1DM and hypovitaminosis D had higher levels of albuminuria compared to those with normal VD levels [albuminuria (log_10_) = 1.92 vs. 1.44; *p* < 0.05].

**Figure 2:**
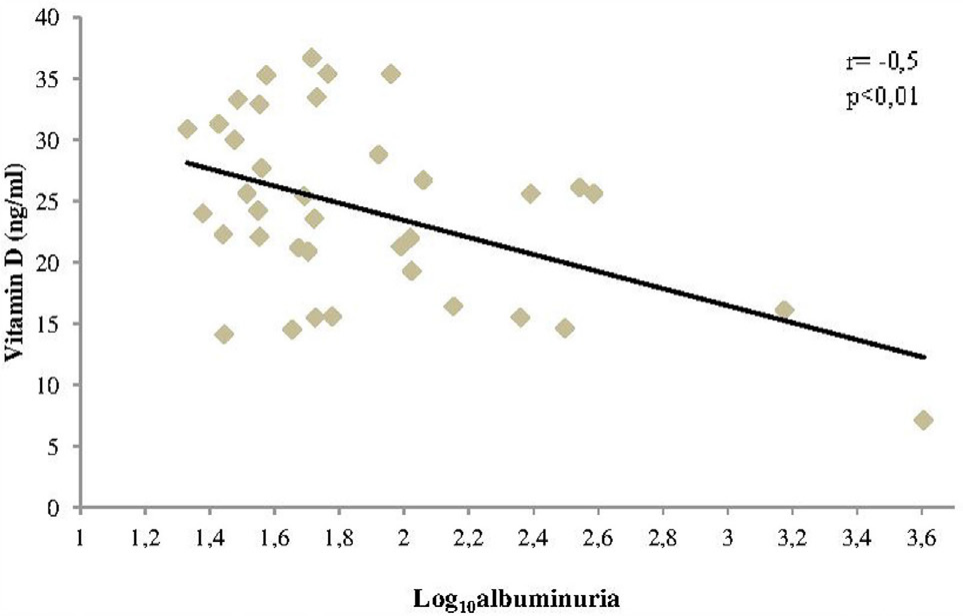
**Correlation between vitamin D levels and albuminuria in patients with T1DM**.

**Figure 3:**
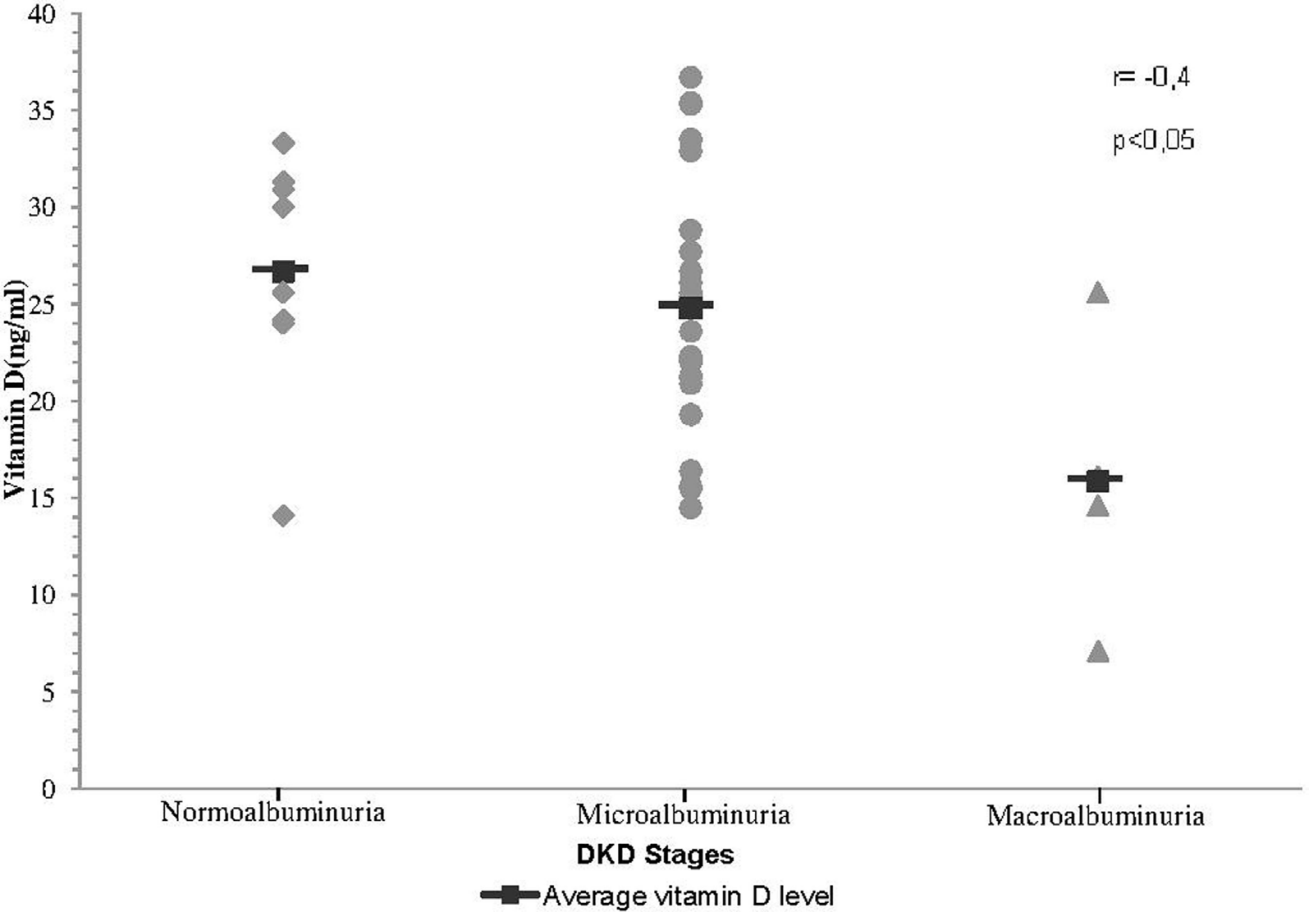
**Correlation between VD levels and stages of DKD**.

**Figure 4:**
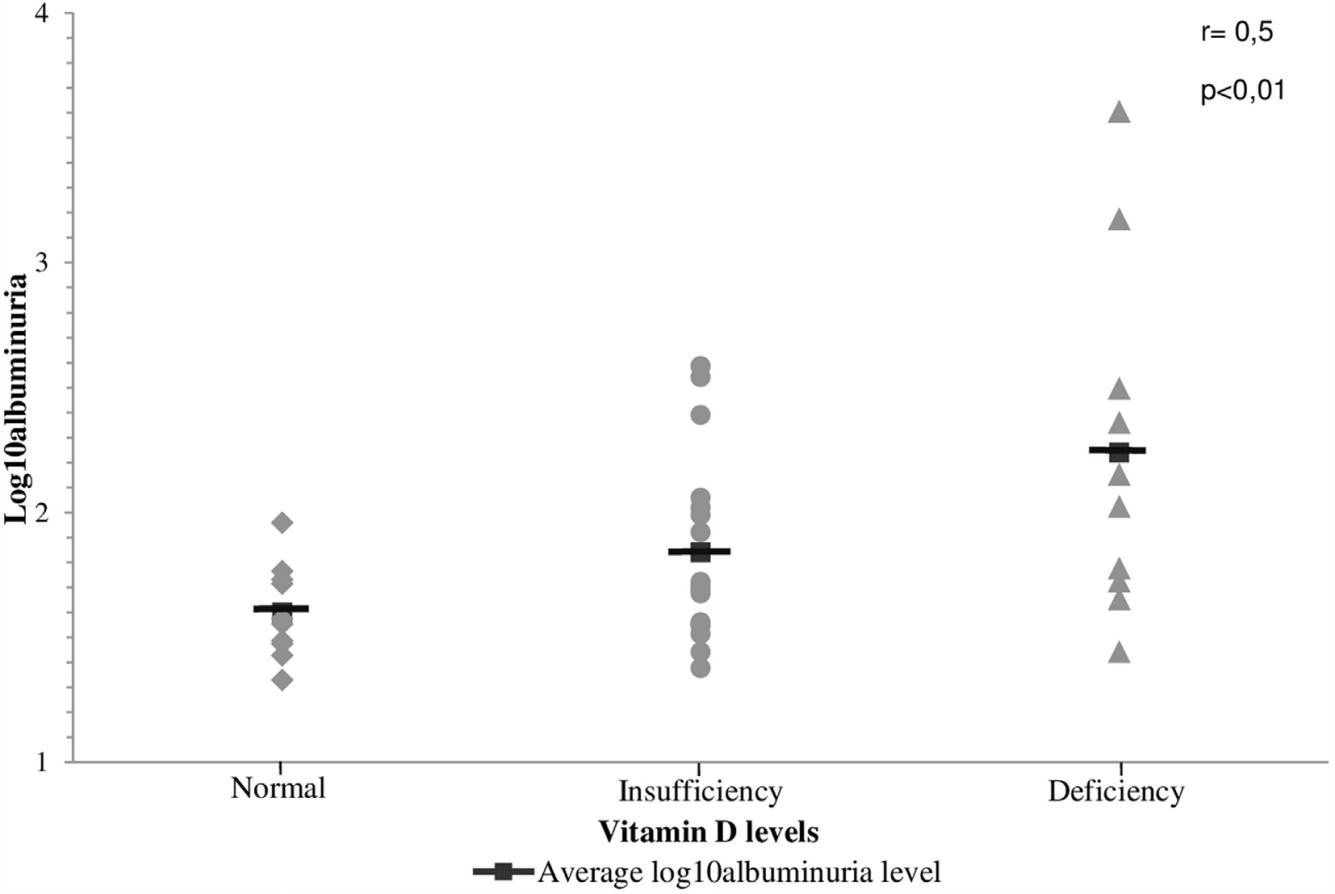
**Correlation between the levels of albuminuria and the status of vitamin D levels in patients with T1DM**.

A correlation was found between VD and albuminuria (transformed into log_10_) in T1DM (Figure 2). In addition, we have found a progressive decrease of VD levels as the stage of DKD worsened (Figure 3). In addition, albuminuria levels according to the status of VD levels had similar behavior (Figure 4).

Our simple linear regression model, with albuminuria as the dependent variable and VD as an independent variable, showed a *r*^2^ = 0.2 and *p* < 0.01. The β1 coefficient was equal to −43. This suggests that every increase of 1 ng/ml of VD could result in a reduction of 43 mg/g in albuminuria.

Our backward stepwise regression model has found that VD, HbA1c, and duration of T1DM were independent predictors of albuminuria. When we have included those three variables in a multiple linear regression model, we have found an increase in predictive value to albuminuria when compared to 25(OH)D alone (*r*^2^ = 0.34 and *p* < 0.01).

## Discussion

Our data suggest an association between reduced levels of VD and the presence and severity of DKD in T1DM. There are few studies (3–5) assessing VD levels in the early stages of DKD and its association with the presence of microalbuminuria with conflicting results. Joergensen et al. (4) in 2011, evaluated VD as a predictor for progression of normoalbuminuria to microalbuminuria in T1DM. To this end, they collected VD before the development of microalbuminuria, and these patients were followed for about 26 years. In this period, 81% of patients developed microalbuminuria, and this was not associated with reduced levels of VD. Differently, Verrotti et al. (3) have compared VD levels and urinary albumin excretion in 22 patients with T1DM and normoalbuminuria, 24 T1DM and microalbuminuria, and 24 controls. Their findings showed lower levels of 25(OH)D in patients with microalbuminuria when compared to other groups. Additionally, de Boer et al. have tested associations between circulating VD metabolites and microalbuminuria in T1DM. They have found that low plasma concentrations of VD (below 20 ng/ml) were associated with an increased risk of microalbuminuria in 65% of patients. Our findings reinforce the data described by de Boer et al. (5). Although there was no difference between the levels of 25(OH)D between T1DM with normo and microalbuminuria, we have demonstrated a good correlation between the reduction in VD levels and evolution of DKD stages.

It has been reported involvement of VD deficiency in the development of diabetes (10). However, it is unclear whether VD deficiency could be an environmental factor involved in the pathophysiology and progression of DKD. The VD receptors (VDRs) are present in the kidney (11). Zhang et al. (12) in 2008 has demonstrated that inactivation of the VDR results in development of more severe DKD in rats, suggesting a renoprotective role of VD against renal injury by regulating the renin–angiotensin system and other genes. In their study, diabetic rats were separated according to presence or not of VDR. After 5 weeks, both groups developed abnormal albuminuria; however, this was more precocious and significant in the group of animals with absent VDR. In addition, WuWong et al. (13) in 2013 demonstrated for the first time that calcitriol exhibits a consistent effect on regulating VDR target gene expression, and calcidiol appears to have differential effects on the expression of different genes. It is not well studied whether the prehormones have effects similar to VDR agonists in providing cardiovascular benefits and/or improving survival in CKD. The VITAL study (14) has showed that treatment for 24 weeks with 2 μg of paricalcitol (VD analog) in patients with type 2 diabetes and DKD results in a reduction on excretion of residual albuminuria. In contrast, a reduction has not been demonstrated in individuals using 1 μg daily, as well as those on placebo. Additionally, it is unclear if an increase of VD levels to >30 ng/ml during a long time could help to elucidate whether VD have possible therapeutic effect on DKD in early stages. Therefore, there are no studies indicating the optimal level of VD supplementation.

A possible role of VD in DKD is the loss of VD carrier proteins (BPD). Thrailkill et al. (15) have analyzed the renal loss of BPD in diabetic patients and have found an increase in the urinary loss of this protein, with a higher increase in patients with abnormal albuminuria, suggesting that this mechanism could collaborate with hypovitaminosis D. Therefore, the question whether the urinary loss of this protein would be a risk marker of renal injury or a causal factor could be raised. Our regression data points to a VD direct action mechanism on urinary albumin excretion independently of diabetic glycemic control.

An interesting study design that could evaluate the role of VD as an environmental factor of DKD could be cohort study in T1DM with normoalbuminuria with evaluation of VD levels to observe the development of DKD. However, this would be an unethical study, due to the need to replace VD when deficiency is detected. The closest way to answer this question would be through the treatment of T1DM with newly diagnosed microalbuminuria, even in the absence of VD deficiency, in an attempt to maintain VD levels above 30 ng/ml and follow the progression of DKD. If this hypothesis is confirmed, a major step would be taken with the use of VD in these cases.

In our study, the prevalence of VD deficiency in T1DM was not different from controls. Pozzilli et al. (16) have demonstrated high VD deficiency rates in patients with newly diagnosed T1DM when compared to controls on a sunny town of Italy in the Lazio region. At the same time, Feng et al. (17) in 2015 conducted a meta-analysis reviewing 13 articles with a total sample of 3494 participants (1790 healthy controls and 1704 with T1DM) and have confirmed the previous data. In this meta-analysis, 25(OH)D levels in T1DM were 2.61 ng/ml lower than controls. Our control group have presented higher BMI when compared to T1DM. It has been reported that excess weight is associated with VD deficiency (18), so it could justify in part the fact that we have found no differences in VD levels between the two groups.

Currently, we have no large studies to determine the prevalence of VD deficiency in Brazil. Data from the city of Recife indicate low levels of VD in postmenopausal women (43%) (10), while Linhares et al. (19) evaluating 226 children of the same population and geographic region have found no VD deficiency. In contrast, in a study with 102 holmes for the elderly in Porto Alegre, where the weather is less sunny, the prevalence of VD deficiency was considerably higher (87.5%) (20). Our region is warm, humid, and sunny; however, our population has the habit to protect yourself from the sun. These factors may have influenced our results and led to a high prevalence of VD deficiency in controls.

Among the weaknesses of our study is the low number of patients with normoalbuminuria when compared to those with microalbuminuria. This fact, associated with the great variability of albuminuria in T1DM, could have been responsible for no differences between VD levels in these two groups. The number of patients with macroalbuminuria also need to be increased. But, the last problem is more difficult to resolve because this kind of patients in general are not in early stages of DKD and do not have normal GFR.

The clinical applicability of our study would be to establish an association between VD levels and DKD in early stages, but the number of patients must be increased. Prospective studies are necessary to establish whether the replacement or supplementation of VD could be useful in the treatment of diabetes kidney disease in T1DM.

## Statement of Human and Animal Rights

All procedures followed were in accordance with the ethical standards of the responsible committee on human experimentation (institutional and national) and with the Helsinki Declaration of 1975, as revised in 2008 (5).

## Statement of Informed Consent

Informed consent was obtained from all patients for being included in the study.

## Author Contributions

RL collected data, and wrote and reviewed the manuscript. FM, FR, AO, AP, JN, CC, DS, MS, NQ, ML, EY, and KF have contributed by creating the database and contacting patients. JF wrote, edited the final version, and was responsible for submitting the manuscript. All the authors read and approved the final manuscript and agreed to its submission.

## Conflict of Interest Statement

The authors declare that the research was conducted in the absence of any commercial or financial relationships that could be construed as a potential conflict of interest.
